# Evaluation of Postural Parathyroid Hormone Change in Patients with Primary Hyperparathyroidism

**DOI:** 10.1155/2014/628010

**Published:** 2014-09-01

**Authors:** Cevdet Aydin, Sefika Burcak Polat, Ahmet Dirikoc, Berna Ogmen, Neslihan Cuhaci, Reyhan Ersoy, Bekir Cakir

**Affiliations:** Endocrinology and Metabolism Department, Ataturk Education and Research Hospital, Yildirim Beyazit University, 06800 Ankara, Turkey

## Abstract

*Purpose*. In the present study, we aimed to investigate postural change of PTH in normal individuals and in patients with primary hyperparathyroidism (PHPT).* Methods*. Twenty-two patients with PHPT and nine healthy controls were enrolled. Following 12 h of fast, patients stayed in recumbent position for an hour and PTH and total Ca measurements were performed at the 45th and 60th minutes of resting. Afterwards, the patients resumed an upright posture for an hour and again blood samples were taken at the 45th and 60th minutes of standing.* Results*. In the PHPT group, mean PTH was calculated as 153.9 pg/mL in the recumbent position while it was 206.3 during upright position (Δ change was 47.7) (*P* < 0.001). In the control group mean serum PTH was measured as 41.2 pg/mL in the recumbent position while it was 44.8 pg/mL in the upright position (Δ change was 1.7) (*P* = 0.11). In both groups, serum Ca was higher in the upright position compared to the recumbent position (*P* < 0.001).* Conclusion*. Postural change of serum PTH is significant only in PHPT group. Postural PTH test may give a clue to the clinician when the diagnosis of PHPT is equivocal.

## 1. Introduction

The major determinants of parathyroid (PTH) secretion are serum calcium (Ca) and vitamin D levels [1]. Serum Ca can vary according to the patient's posture prior to obtaining a blood sample [2], and the effect of posture on serum Ca levels has been demonstrated previously. Serum Ca follows a diurnal rhythm [3–6]. Normally, in the upright position, intravascular fluid ultrafiltrates into the interstitial space, thereby increasing the protein content of plasma and the concentration of protein-bound molecules such as serum total Ca [7].

Diurnal variation in PTH levels was demonstrated as early as the 1960s [8–10] and has been confirmed in recent studies using the intact PTH assay, with peak levels occurring during the late hours of the evening [11, 12]. However, it remains unclear whether this nocturnal increase in PTH is part of a circadian rhythm or if it depends on other factors such as posture or sleep.

A change in posture, from the recumbent to upright position, leads to an increase in the plasma levels of several hormones, including cortisol, prolactin, renin, and aldosterone [13, 14].

Primary hyperparathyroidism (PHPT) is characterized by inappropriately elevated or normal PTH in presence of hypercalcemia. The causes of hypercalcemia that result in elevated parathyroid hormone level are few including familial benign (hypocalciuric) hypercalcemia (FHH), lithium-induced hypercalcemia, and tertiary hyperparathyroidism. A subset of patients had calcium levels within the reference range with elevated parathyroid hormone, so-called normocalcemic hyperparathyroidism [15].

We investigated the relationship between postural changes and PTH levels in patients with primary hyperparathyroidism. To our knowledge, no previous study has assessed this relationship in the context of recumbent-to-upright postural changes, in either healthy controls or primary hypoparathyroidism (PHPT) patients.

## 2. Subjects and Methods

### 2.1. Subjects

Twenty-two patients (five males and 17 females; mean age, 50.7 ± 10.9 years) with primary hyperparathyroidism were enrolled in the study. The diagnostic criteria for PHPT were as follows: high serum calcium levels (>10.5 mg/dL), inappropriately elevated or normal PTH levels (in the presence of normal renal function), and normal or high levels of 24-hour urinary calcium excretion. Nine healthy volunteers (four males and five females; mean age, 28.9 ± 5.2 years) comprised the control group.

Healthy volunteers demonstrated normal medical histories and results from physical examination and laboratory studies (i.e., no chronic diseases which could affect PTH, vitamin D, or calcium metabolism).

The study was reviewed and approved by the Committee for the Protection of Human Subjects, part of the local ethics committee of our university. Informed consent was provided by each subject prior to their participation.

### 2.2. Study Protocol

Following a 12 h fast, a cannula was placed into the antecubital vein of all subjects, who were then asked to remain in the recumbent position for 1 h, following the fulfilment of any physical needs such as urinating. The moment at which the patient lied down corresponded to “0 min.” Blood was withdrawn at 45th and 60th min from subjects in the recumbent position, to allow for measurement of PTH and Ca from the i.v. line, without the need for a new venipuncture or potential additional stress. Following pulse and blood pressure measurements, each patient was requested to resume an upright posture. The moment at which the patient stood up corresponded to “0 min;” blood pressure and pulse rate were then measured again. For subjects in the standing position, blood was again withdrawn at the 45th and 60th min, to allow for measurement of PTH and Ca. Following 1 h of standing, the test was terminated. To achieve standardization, testing commenced between 9:00 and 9:30 A.M. for all subjects.

### 2.3. Laboratory Tests

Total calcium was determined using a reference clinical chemistry laboratory (8.8–10.2 mg/dL) (Roche Diagnostics, Manheim, Germany).

Plasma intact PTH was measured using the Allegro immunoradiometric assay (Roche Diagnostics, Manheim, Germany). The detection limit of the assay was 1 pg/mL (normal range, 10–65 pg/mL), and the intra- and interassay coefficients of variation were 2% and 10%, respectively. Vitamin D was measured by liquid chromatography coupled with tandem mass spectrometry (Schimadzu-API LC-MS-MS API 3200, Canada). The lower and upper detection limits were 4 and 150 *μ*g/L, respectively.

### 2.4. Statistical Analysis

All analyses were performed using the SPSS for Windows software package (ver. 11.5, IBM Corp, Armonk, NY, USA). The Shapiro-Wilk test was used to test the normality of continuous and other variables. Descriptive statistics are presented as means ± SD, with medians (minimum-maximum) for continuous variables and percentages (%) for categorical variables. Group differences in means were compared using Student's* t*-test. Median values were compared using the Mann-Whitney* U* test. For categorical variables, differences were assessed using Fisher's exact test.

Values obtained in the same subjects in the recumbent and upright positions were compared by Wilcoxon's test. The significance of difference between Ca measurements was evaluated with dependent* t*-test. Differences between Ca measurements were evaluated using dependent* t*-tests. A value of *P* < 0.05 was taken to indicate statistical significance.

## 3. Results

Patients were significantly older than controls (*P* < 0.001), but sex distribution did not differ between the two groups (*P* = 0.385). Body mass index (BMI) was significantly higher in patients compared with controls (*P* < 0.001). 25 OH vitamin D level did not differ between groups (*P* = 0.053); however, when a value of 75 *μ*g/L, present in the patient group, was omitted from the analysis, the group difference reached significance (*P* = 0.025; Table 1). Seventeen of the twenty-two patients diagnosed with PHPT underwent surgery and received a final diagnosis of single parathyroid adenoma; one patient was diagnosed with parathyroid hyperplasia. Four PHPT patients were asymptomatic and did not meet the indications for the surgery criteria; one patient did not consent to surgery even though she was symptomatic for osteoporosis.

Among the 17 patients who underwent surgery, seven (41.2%) were tested positive for nephrolithiasis and 10 (58.8%) for osteoporosis. The most commonly reported symptom was weakness (*n* = 6). One patient was constipated, another reported severe nausea, and three others experienced generalized bone pain. Finally, one patient reported numbness of the tongue, and five were completely asymptomatic for hypercalcemia.

There was no significant difference in PTH levels, measured at 45 and 60 min with subjects in the recumbent position, for the control (*P* = 0.314) or patient group (*P* = 0.242). This was also the case when the subjects were measured in the standing position, for both the control (*P* = 0.374) and patient groups (*P* = 0.256).

Control group serum Ca levels were higher at 45 versus 60 min in the recumbent position (*P* = 0.014), but no difference was observed between 45 and 60 min serum Ca levels in the patient group (*P* = 0.429).

Serum Ca measurements at 45 versus 60 min in the standing position did not differ between the control (*P* = 0.400) and patient groups (*P* = 0.183).

**Figure 1:**
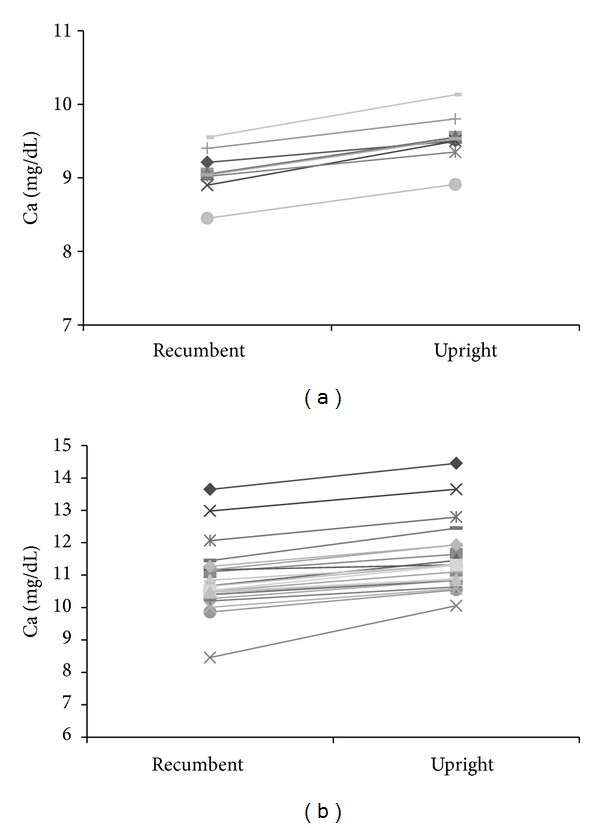
Serum Ca response to the upright posture for each subject in the control (a) and patient group (b).

Mean Ca levels were significantly higher in the upright than the recumbent position for both the control (*P* < 0.001) and patient groups (*P* < 0.001; Table 2). On Figures 1(a) and 1(b), response to upright position is depicted for each subject for Ca.

**Figure 2:**
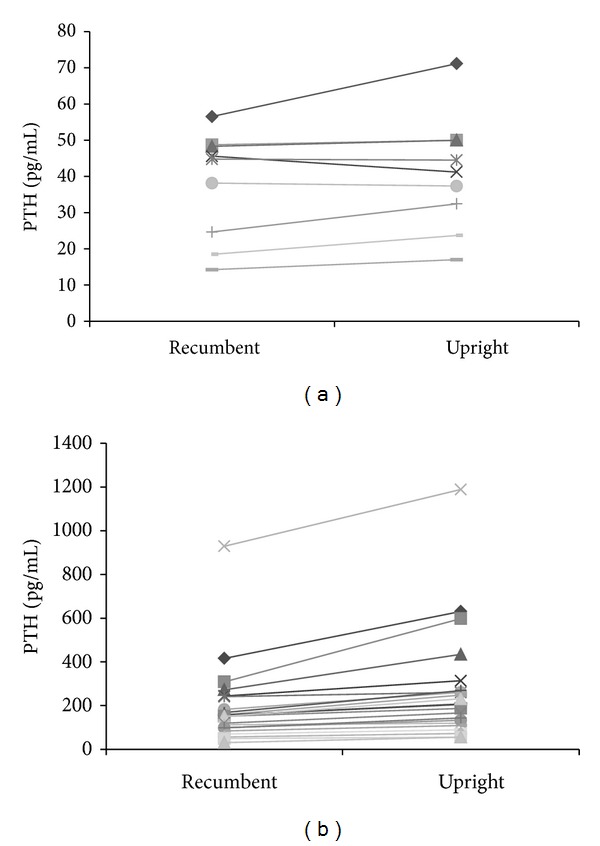
Serum PTH response to the upright posture for each subject in the control (a) and patient group (b).

There was no significant difference in mean PTH levels between the upright and recumbent positions for the control group (*P* = 0.110). However, in the patient group, upright position mean PTH levels were significantly higher compared with recumbent position mean PTH levels (*P* < 0.001; Table 2). On Figures 2(a) and 2(b), response to upright position is depicted for each subject for PTH.

The degree of incremental change (i.e., delta change) in the PTH level when posture changed from the recumbent to upright position was greater in patients compared with controls (*P* < 0.001). In contrast, incremental serum Ca changes, in accordance with postural changes, did not differ between groups (*P* = 0.065; Table 3). When we considered the percentage of increment in the PTH level in accordance with recumbent to upright postural changes, we observed more prominence in the patient group compared with the control group. There was no group difference in percentage change of serum Ca between the different postures (*P* = 0.334; Table 3).

In the control group, mean systolic and diastolic blood pressure measured in the recumbent position were 102.2 ± 13.01 and 63.3 ± 5 mm Hg, respectively, compared with 98.8 ± 15.3 and 64.4 ± 5.27 mm Hg in the upright position. The mean pulse rate was 73 ± 6.54 per min in the recumbent position, compared with 84.33 ± 9.24 per min in the upright position.

In the PHPT group, mean systolic and diastolic blood pressure measured in the recumbent position were 119.7 ± 14.76 and 75 ± 9.86 mm Hg, respectively, compared with 118.68 ± 14.98 and 74.47 ± 9.26 mm Hg in the upright position. The mean pulse rate was 75.88 ± 7.11 per min in the recumbent position, compared with 78.83 ± 9.53 per min in the upright position.

## 4. Discussion

PTH levels did not differ according to recumbent-to-upright postural changes in controls, whereas they increased significantly in the PHPT patients. Serum Ca levels also changed significantly in accordance with postural changes in both groups and were significantly higher in the upright position. Repeated PTH and Ca measurements (at 45 and 60 minutes) in each posture and for both groups were performed.

In a standing position, fluid normally leaves the intravascular compartment, probably due to an increase in hydrostatic pressure. As a result, larger proteins and protein-associated substances do not easily cross between compartments, and accordingly, their concentrations increase. Therefore, for outpatients, the time that the patient spends in the sitting position before the serum sample is collected may have a significant effect on total calcium values [2]. The expected change in serum total Ca is approximately 4.6%. According to the results of our study, serum Ca increased by 5.4% in controls and 5.7% in patients, which is in accordance with previous studies [2, 7].

Few studies have been conducted on the circadian rhythm of PTH [16], and no studies have been concerned with the effects of postural changes on PTH.

PTH levels increased significantly in response to postural change in the patient group, with increased levels observed in the upright position. It can be suggested that increased protein concentrations in response to assuming the upright position could result in decreased levels of ionized calcium, which are not captured by albumin adjustment.

There are several other possible mechanisms which could explain the significant changes in PTH levels in response to postural changes in primary hyperparathyroidism. First, the response of parathyroid adenoma cells to autonomic innervation differs to that of normal parathyroid cells. Second, parathyroid adenoma cells might have calcium-sensing receptors, the responses of which change according to posture. PTH secretion is closely controlled by ionized Ca concentrations, through the regulatory activity of calcium-sensing receptors (CaSRs) [17, 18]. In recent studies, CaSR expression in parathyroid glands (PTGs) was shown to decrease in cases of primary hyperparathyroidism, as measured by several different techniques including immunohistochemistry [19].

Another mechanism that could underlie significant changes in PTH levels in PHPT patients in accordance with postural changes might involve mutual interactions between aldosterone and PTH. It has been hypothesized that aldosterone affects calcium metabolism, and that human osteoblasts and osteoclasts possess mineralocorticoid receptors [20]. Furthermore, PTH levels are higher in patients with primary hyperaldosteronism [21]. Rossi et al. (1995) documented higher levels of serum PTH and lower serum calcium concentrations in patients with primary aldosteronism (PA) compared with patients with essential hypertension. Spironolactone treatment was associated with higher serum calcium concentrations and lower serum PTH levels [22]. However, these data do not explain why increased aldosterone, observed in the upright position, was associated with a greater increase in PTH in PHPT patients compared with controls; therefore, further studies are required. In another study, serum PTH levels increased after dialysis, due to the occurrence of hypovolemia following ultrafiltration; the authors postulated that extracellular volume depletion and central hypovolemia exerted an effect independent of serum calcium on plasma PTH concentrations in dialysis patients [23].

Because we did not measure serum ionized Ca and aldosterone, any explanations for the possible mechanisms underlying PTH changes in PHPT patients in response to postural changes remain speculative. The only parameters of autonomic regulation that we evaluated were arterial blood pressure and pulse rate, which did not differ between the recumbent and upright positions in PHPT patients.

A limitation of our study was the small sizes of the PHPT patient and control groups. However, we increased the validity of our study by taking two measurements, for both PTH and Ca, in each posture and in both groups. A second limitation concerned the lack of ionized Ca measurements, which would have been particularly useful since ionized Ca is the main regulator of PTH synthesis.

In conclusion, serum Ca is affected by postural changes, whereas changes in serum PTH levels in response to postural changes were only significant in PHPT patients and not in controls. Further studies using larger sample sizes are required to elucidate the potential for using postural PTH measurements during PHPT diagnosis. In addition, postural parathormone tests might be useful if the PHPT diagnosis is equivocal, as is frequently observed with normocalcemic PHPT, the prevalence of which continues to increase.

## Figures and Tables

**Table 1:** Demographic characteristics of the patient and control groups.

Variables	Control group	Patient group	*P* value
Age (years)	28.9 ± 5.2	50.7 ± 10.9	<**0.001**
Female/male	5/4 (55.6/44.4%)	17/5 (77.3/5%)	0.385
BMI (kg/m^2^)	20.7 ± 3.0	28.0 ± 4.8	<**0.001**
Vitamin D (*μ*g/L)	27.9 (8.8–35.4)	10.8 (7.9–75.0)	0.053∗

*P = 0.025 when a value of 75 *μ*g/L, present in the patient group, was omitted, and the Mann Whitney *U* test was applied for reevaluation.

**Table 2:** Comparison of mean Ca and PTH levels measured in 2 different postures within the control and patient groups.

Variables	Recumbent	Upright	*P* value
PTH (pg/mL)			
Control goup	44.8 (14.2–56.5)	41.2 (17.0–71.2)	0.110
Patient group	153.9 (31.2–929.0)	206.3 (55.2–1189.5)	<**0.001**
Ca (mg/dL)			
Control group	9.07 ± 0.31	9.54 ± 0.33	<**0.001**
Patient group	10.84 ± 1.06	11.49 ± 1.05	<**0.001**

**Table 3:** Comparison of the control and the patient groups according to amount of postural change in serum PTH and Ca.

Variables	Control group	Patient group	*P* value
Delta PTH	1.7 (−4.4–14.6)	47.7 (5.9–289.3)	<**0.001**
Delta Ca	0.46 ± 0.10	0.65 ± 0.28	0.065
PTH change (%)	3.5 (−9.6–31.6)	33.3 (6.8–93.7)	<**0.001**
Ca change (%)	5.4 (3.1–6.7)	5.7 (1.4–18.9)	0.334
